# Deciphering the Role of Host Genetics in Susceptibility to Severe COVID-19

**DOI:** 10.3389/fimmu.2020.01606

**Published:** 2020-06-30

**Authors:** Madalina Elena Carter-Timofte, Sofie Eg Jørgensen, Mette Ratzer Freytag, Michelle Mølgaard Thomsen, Nanna-Sophie Brinck Andersen, Ali Al-Mousawi, Alon Schneider Hait, Trine H. Mogensen

**Affiliations:** ^1^Department of Biomedicine, Aarhus University, Aarhus, Denmark; ^2^Department of Infectious Diseases, Aarhus University Hospital (AUH), Aarhus, Denmark; ^3^Department of Clinical Medicine, Aarhus, Denmark

**Keywords:** SARS-coronavirus 2, host immune defenses, immunopathology, innate immunity, primary immunodeficiency, whole exome sequencing

## Abstract

Coronavirus disease-19 (COVID-19) describes a set of symptoms that develop following infection by the severe acute respiratory syndrome coronavirus 2 (SARS-CoV-2). Whilst COVID-19 disease is most serious in patients with significant co-morbidities, the reason for healthy individuals succumbing to fulminant infection is largely unexplained. In this review, we discuss the most recent findings in terms of clinical features and the host immune response, and suggest candidate immune pathways that may be compromised in otherwise healthy individuals with fulminating COVID-19. On the basis of this early knowledge we reason a potential genetic effect on host immune response pathways leading to increased susceptibility to SARS-CoV-2 infection. Understanding these pathways may help not only in unraveling disease pathogenesis, but also in suggesting targets for therapy and prophylaxis. Importantly such insight should instruct efforts to identify those at increased risk in order to institute preventative measures, such as prophylactic medication and/or vaccination, when such opportunities arise in the later phases of the current pandemic or during future similar pandemics.

## Introduction

In late December 2019, several cases of acute respiratory distress syndrome (ARDS) were reported across several provinces in China, and by March 2020, the World Health Organization (WHO) declared the current outbreak a global pandemic (1, 2). Six zoonotic coronaviruses are known to have the capability to cause respiratory disease in humans. The human coronaviruses (HCoV) 229E, NL63, OC43, and HKU1 are identified as weakly pathogenic, causing mild upper respiratory disease (3). However, during the last two decades, public health has been threatened by two highly pathogenic coronaviruses, including severe acute respiratory syndrome coronavirus (SARS-CoV) in 2002, and Middle-East respiratory coronavirus, (MERS-CoV) in 2012 (4). The 2019/20 outbreak was rapidly identified to be caused by a new member of the coronavirus family, namely SARS-CoV-2. This virus spreads by human-to-human transmission and early observational data has suggested a reproductive number of 2.2 days. Together with a mean incubation time of 5.2 days, SARS-CoV-2 has the ability to spread widely among humans (5). As of April 2020, ~8.9 million cases of coronavirus diseases (COVID-19) and 465,740 deaths have been reported globally according to the WHO. Whilst the majority of deaths have been of elderly individuals or patients with underlying health conditions, a small number of young and healthy people have also succumbed to fulminant infection. In these rare cases, host genetics may provide some explanation for failure to control SARS-CoV-2 infection.

Primary immunodeficiencies (PIDs) are a group of genetically determined diseases predisposing individuals to severe infection, immune dysregulation, autoimmunity and malignancy (6, 7). There is increasing evidence that the extreme variability in the clinical outcome of infections can often be influenced by the germ line genetics of the human host (8). Such monogenic inborn errors of immunity predispose to a narrow or broad range of viral infections (6, 9, 10). Examples include herpes simplex virus encephalitis and defects in the TLR3 pathway and RNA metabolism (9, 11, 12), varicella-zoster virus and defective innate RNA POL III signaling (13–15), severe influenza and defects in IRF7 (16), IRF9 (17), TLR3 (18), GATA2 (19), and RIG-I (20), rhinovirus infection and MDA5 (21), papillomaviruses and mutations in EVER1/2 (22), EBV lymphoproliferation and malignancy in the case of defects in SH2D1A (23), CD27, CD70 (24), XIAP (25), ITK (26), and MAGT1 (27), fatal CMV infection in NOS2 deficiency (28), and finally disseminated infection with measles vaccine strain virus in IFNAR and STAT2 deficiency (29, 30). In this review, we aim to discuss the most recent findings in terms of the innate and adaptive immune response to SARS-CoV-2, and draw on knowledge from the previous SARS-CoV and MERS-CoV outbreaks where relevant. This knowledge should enable us to speculate and to suggest essential antiviral pathways that may be defective in individuals at increased risk of severe COVID-19 and thus suggest genetic etiologies which may predispose otherwise healthy individuals without comorbidities to severe SARS-CoV-2 infection.

## Clinical Presentations of COVID-19

The severity of COVID-19 is extremely variable, with some individuals reporting almost no symptoms, whereas others become critically ill requiring intensive care and respiratory ventilation (31–33). The frequency of asymptomatic infections is yet to be determined and the differentiation between those and pre-symptomatic cases should be considered (34, 35). In the largest case report of 44,672 confirmed cases of COVID-19 described by the Chinese CDC, an overall case fatality rate (CFR) of 2.3% was calculated (36). However, the mortality rates vary widely between different ages. No deaths were reported in children <9 years old, but the CFR increases to 14.8% in the 80+ age group. In critical cases requiring ventilator support in the ICU, a CFR of 49–61% was reported (36). Whilst severe disease outcomes have been reported in otherwise healthy individuals of any age, several risk factors are recognized that lead to a more critical disease course; these include old age, hypertension, diabetes, obesity, cardiovascular, pulmonary, and cerebrovascular disease (37).

COVID-19 initially presents with non-specific signs and symptoms of upper airway viral infection characterized by fever, fatigue, cough, and dyspnoea, as well as anorexia, myalgia, and productive sputum, which can develop to pneumonia (32, 38). Intriguingly, patients are increasingly reporting anosmia and dysgeusia, the loss of smell and taste, reflecting some neurological effect of SARS-CoV-2 (39), which has subsequently been confirmed by several reports of central and peripheral nervous system manifestations, with meningitis, encephalitis, myelitis, and Guillain-Barre syndrome presenting as components of severe COVID-19 (40, 41). In this context, respiratory failure has been hypothesized to be partly neurogenic in origin and possibly resulting from viral invasion of cranial nerve I into brainstem respiratory centers (41). Gastrointestinal symptoms, particularly nausea, vomiting, and diarrhea, are commonly described (42). In the most critical cases, widespread lung inflammation can lead to acute respiratory distress syndrome (ARDS), which often necessitates mechanical ventilation or extracorporeal membrane oxygenation (ECMO) to prevent total respiratory failure (43). Recent studies have demonstrated an extensive tendency for coagulopathy, thrombosis, micro-thrombosis, and disseminated intravascular coagulation during severe COVID-19 (44, 45) which is reflected in elevated fibrin and D-dimer levels during disease (46). Furthermore, a significant fraction of deceased ICU patients have pulmonary thrombosis or deep vein thrombosis as revealed by autopsy results (47).

Several clinical and immunological studies have suggested that excessive inflammation and a cytokine storm play a key role in the immunopathology, responsible for much of the lung damage, morbidity and mortality in patients with severe COVID-19 in ICUs (48–50). This concept opens up the possibility that medical treatments that dampen immune activation may represent an important strategy in treating this disease and decreasing the high mortality (51, 52). Finally, there is emerging evidence that some infants and children have presented with a clinical picture of auto-inflammation and vasculitis, diagnosed as atypical Kawasaki's disease (53), a rare disease entity causing systemic inflammation, fever, and vasculitis with a risk of developing aneurisms. This inflammatory condition associated with a previous SARS-CoV-2 infection has been named Pediatric Multisystem Inflammatory Syndrome temporally associated with SARS-CoV-2 (PIMS-TS) (54).

## SARS-CoV-2

Coronaviruses are enveloped, positive-sense, single-stranded viruses that contain the largest genome amongst RNA viruses (27–32 kb) (55, 56). SARS-CoV-2 belongs to the *Betacoronavirus* genera, based on its phylogenetic relationship with other *Betacoronaviruses*, such as SARS-CoV and MERS-CoV (57, 58). Whilst SARS-CoV-2 shares 79% nucleotide sequence identity with SARS-CoV, Zhou et al. reported the SARS-CoV-2 sequence to be 96.2% identical to the sequence of the horseshoe bat coronavirus, RaTG13, suggesting that SARS-CoV-2 originates from this bat species (58). An intermediate animal host between horseshoe bats and humans, as well as the transmission route, has yet to be identified in the SARS-CoV-2 pandemic (59). However, the most prevalent hypothesis is that the virus acquired one or several mutations allowing it to cross species barriers and infect human cells some time in autumn 2019, a few months before the beginning of the current pandemic (60).

Like other coronaviruses, the major structural proteins of SARS-CoV-2 include the envelope (E), membrane (M) and spike (S) proteins (61). In the earlier SARS-CoV epidemic, the S protein was shown to facilitate cellular entry by binding to the angiotensin-converting enzyme 2 (ACE2) receptor present on target cells (62). In addition, the cellular serine protease transmembrane protease serine 2 (TMPSS2) is needed for priming of the S protein and subsequent fusion of SARS-CoV with the host cell membrane (63, 64). Furthermore, overexpression of ACE2 enhances disease severity in mice upon SARS-CoV infection, indicating the importance of this receptor in facilitating viral entry (65). Several studies have shown that the S protein of SARS-CoV-2 also uses ACE2 and TMPRSS2 for cell entry, and that the SARS-CoV-2 S protein is able to bind to ACE2 with 10- to 20-fold higher affinity than the SARS-CoV S protein (66–69). Furthermore, ACE2 is highly expressed by type II alveolar epithelial cells (70, 71) which correlates with the pulmonary symptoms of COVID-19 (32). However, ACE2 is also highly expressed by intestinal, heart, kidney and bladder cells (71, 72). This pattern of cellular ACE2 expression may explain some of the non-respiratory symptoms and complications that SARS-CoV-2 patients exhibit, such as diarrhea, kidney failure and cardiac injury (32, 73).

## Innate Immune Responses to SARS-CoV-2

**Figure 1:**
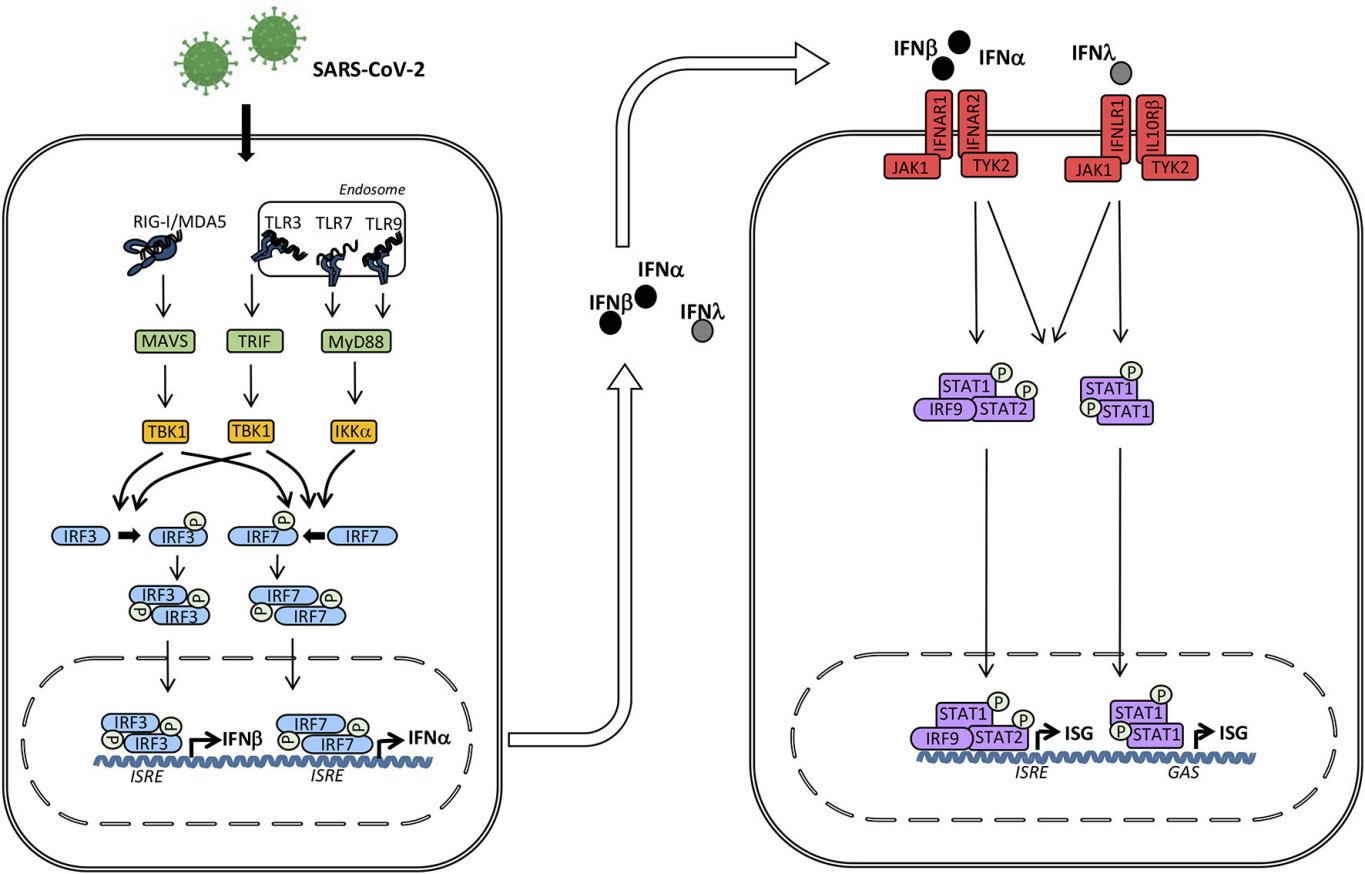
Induction of interferons (IFN) and signaling by the type I and III IFN receptors. The presence of microbial or self nucleic acid in the cytosol or within the endosomal compartment activates pattern recognition receptors (PRR)s. RNA activates retinoic acid-inducible receptor (RIG)-I in the cytosol and Toll-like receptor (TLR)3 and TLR7 in the endosomal compartment. These events trigger signaling pathways through the adaptor molecules mitochondrial antiviral signaling protein (MAVS), TIR-domain-containing adapter-inducing interferon-β (TRIF), and Myeloid differentiation primary response (MyD)88 leading to phosphorylation and activation of the TANK binding kinase (TBK)1, which in turn phosphorylates the transcription factors IFN regulatory factor (IRF)3 and IRF7. Whereas IRF3 is constitutively present, IRF7 is only expressed at low levels but may be secondarily induced by Type I IFN. Phosphorylation of IRF3 and IRF7 leads to homodimerization, nuclear translocation, and expression of Type I IFNs (IFNα and IFNβ) and type III IFNs (IFNλ) acting on neighboring cells with type I and III IFN receptors, respectively. Type I IFN binds to IFNα/β receptor composed of IRFNAR1 and IFNAR2, whereas type III IFN binds to the IFNλ receptor composed of IFNLR1 and IL10Rβ. These events activate the downstream receptor-associated Janus-associated kinase (JAK)1 and tyrosine kinase (TYK)2 and subsequent tyrosine phosphorylation of STAT1 and STAT2. These activated transcription factors together with IRF9 form the heterotrimeric transcription factor IFN-stimulated gene factor (ISGF)3 complex which binds to IFN-stimulated regulatory elements (ISRE) in DNA. In addition, STAT1 homodimers form the IFN-γ-activated factor (GAF) complex which binds to γ-activated (GAS) sequences. Altogether, these transcription factors induce a broad spectrum of IFN-stimulated genes (ISG)s that mediate the complex “antiviral state” of IFNs.

The innate immune response is the first line of defense against a wide range of pathogens, particularly viruses. Importantly, viral infection induces type I interferons (IFN) (IFNα/β) and type III IFNs (IFN-λ), which activate hundreds of antiviral proteins as well as mediate priming the adaptive immune response (Figure 1). Coronaviruses, and their nucleic acid genome and replication intermediates in particular, are recognized primarily by two groups of pattern recognition receptors, namely the Toll-like receptors (TLRs) and the retinoic acid inducible gene I (RIG-I)-like receptors (RLRs). These receptors recognize several viral components, which induce downstream signaling and result in production of antiviral type I and III IFNs as well as proinflammatory cytokines through activation of the transcription factors interferon regulatory factor (IRF)3, IRF7 and nuclear factor(NF)-κB (74) (Figure 1).

**Figure 2:**
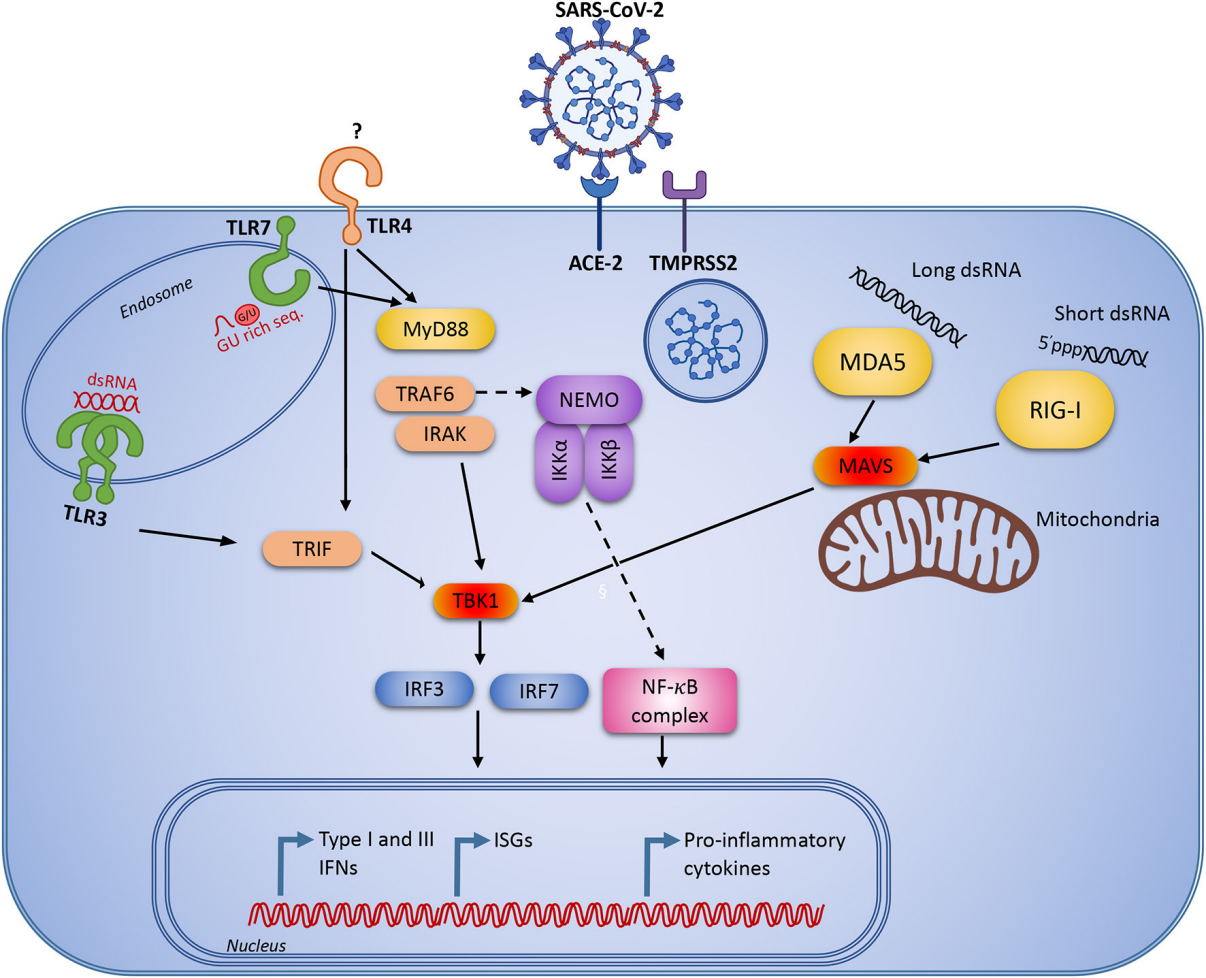
Innate immune signaling pathways that are known to be activated during viral infection and replication. Severe acute respiratory syndrome coronavirus 2 (SARS-CoV-2) infects human cells through the angiotensin-converting enzyme 2 (ACE2 receptor) and the cellular serine protease TMPRSS2 for viral S protein priming. Following cellular entry, the virus RNA genome may be recognized by pattern recognition receptors (PRR)s, including endosomal Toll-like receptor (TLR)3 and TLR7 recognizing double-stranded and single-stranded RNA, respectively. In the cytosol the virus may be recognized by the retinoic acid inducible receptor (RIG)-I or the melanoma differentiation-associated protein (MDA)5. Following viral recognition by PRRs, this triggers signaling through IFN regulatory factor (IRF)3 and NF-κB to induce IFNs and pro-inflammatory cytokines. Similar responses may be activated by extracellular virus through TLR4.

The TLRs which have so far been implicated in the response to SARS-CoV, are TLR3, -4 and -7 (Figure 2). TLR3 recognizes double-stranded RNA (dsRNA), and the coronavirus-derived ligand is likely a double-stranded replication intermediate. The ligand for TLR4 remains obscure, as TLR4 usually recognizes lipopolysaccharide from gram negative bacteria. Finally, special GU-rich sequences in the SARS-CoV genome activate TLR7 (75). TLR3 and TLR4 activate the adaptor TIR-domain-containing adapter-inducing interferon-β (TRIF), whereas myeloid differentiation primary response 88 (MyD88) is the adaptor used by all other TLRs (76) (Figure 2). Insights from mouse knock-out studies may provide some knowledge as to which signaling pathways are important in SARS-CoV-2 recognition, and where we might expect to identify genetic variants, which predispose to more severe COVID-19 disease.

Several studies have demonstrated that infection of *Tlr3*^−/^^−^, *Tlr4*^−/−^*, Trif*^−/−^*, Tram*^−/−^, and *Myd88*^−/−^ mice with SARS-CoV causes increased viral replication, enhanced pathology in the lungs, and increased morbidity (77–79). The most severe phenotypes and mortality are generally seen in mice lacking the downstream adaptors TRIF and MyD88 (77, 78). Interestingly, following SARS-CoV infection of *Trif*^−/−^ mice, the aberrant proinflammatory cytokine and chemokine responses were similar to those seen in human patients with poor disease outcome during SARS-CoV infection (80) or MERS-CoV infection (81). This may suggest that the initial impaired control of viral replication could lead to exaggerated immune responses and enhanced immunopathology later during infection. However, MyD88 and IRAK4 seem to be largely redundant in antiviral immunity in humans, since previous studies have failed to report increased susceptibility to severe viral infection in patients deficient in these molecules (82). This underlines the value of studying the spectrum of infections in patients with well-defined immunodeficiencies *in natura*, revealing important lessons on protective immunity in humans (10, 83).

Coronaviruses are, in addition to being recognition by TLRs, also recognized by RLRs. The RLRs are cytosolic RNA sensors, where RIG-I recognizes short dsRNA with 5' triphosphate, and melanoma differentiation-associated protein 5 (MDA5) recognizes long dsRNA. MDA5 is clearly protective in mice infected with the murine coronavirus mouse hepatitis virus (MHV) (84), whereas there are only limited reports indicating that the other RLRs are important in SARS-CoV infection. This is, however, not due to the virus failing to be recognized by the receptors, but rather because SARS-CoV, as well as other coronaviruses, employs several strategies to avoid recognition and immune activation. For example, deletion of the SARS-CoV nsp16 gene, an enzyme which catalyzes ribose 2'-O-methylation to generate caps on the coronavirus RNA (85), attenuates the pathogenicity of the virus (86, 87). However, in the absence of MDA5 or Interferon Induced Protein With Tetratricopeptide Repeats 1 (IFIT1), the SARS-CoV nsp16 deletion mutant reached similar levels of viral replication and virulence as wild type SARS-CoV (86, 87), indicating that MDA5 recognizes partly uncapped SARS-CoV RNA. The recognition of SARS-CoV by RIG-I has not yet been described, probably due to the fact that RIG-I has a preference for short dsRNA and also because the initial steps of capping impairs RIG-I recognition by removing the 5'triphosphate (85).

Investigations into the role of lung inflammation during respiratory infection identified a potential role for excessive inflammasome activation. Mice in which the inflammasome pathway had been deleted by CASP1/11 knock-out displayed less mortality upon influenza infection compared to wild-type mice (88). Furthermore, inflammation was shown to be driven by excessive neutrophil activation. Given that neutrophilia is a paraclinical indication of COVID-19 disease severity, we might predict that inhibiting this response may improve outcomes in SARS-CoV-2 infection. These studies are supported by other data demonstrating that the NACHT, LRR and PYD domains-containing protein 3 (NLRP3) inflammasome activation by the SARS-CoV viral proteins, E, ORF-8b and viroporin 3a (89–91). Importantly, coronaviruses can induce pyroptosis via NLRP3 in a process involving insertion of Gasdermin D in the cellular membrane (92). Furthermore, several publications have implicated neutrophils and neutrophil extracellular traps (NET) in organ damage, pulmonary pathology, micro-thrombosis, and mortality in COVID-19 (93, 94). NETs consist of extracellular webs of chromatin, microbicidal proteins and oxidant enzymes that are released by neutrophils to contain microbial infections but also have the potential to propagate inflammation and immunopathology (94).

IFNs are the most prominent antiviral effectors of the innate immune system, and SARS-CoV infection is highly susceptible to this potent antiviral substance, as shown by the efficient inhibition of viral replication upon administration of exogenous IFN both *in vitro* and *in vivo* (95–100). However, only very limited amounts of IFNs are actually produced during SARS-CoV and MERS-CoV infections (101), and the response seems to be delayed relative to the production of proinflammatory cytokines (102). However, it is worth noting that these anti-viral responses are most likely cell-type specific. A recent study demonstrated that SARS-CoV-2 also induces significantly less alveolar cell IFN and ISG expression compared to influenza A and respiratory syncytial virus *in vitro* (103). In support of these data, a comparative study reported more efficient viral replication and a reduced type I IFN response in SARS-CoV-2 infection of *ex-vivo* human lung cultures compared to SARS-CoV (104). To achieve this very low IFN production, the coronaviruses are known to employ numerous strategies to counteract the innate immune signaling pathways of the host, which have been reviewed elsewhere (101, 105–107). However, the exact mechanisms by which SARS-CoV-2 appears to more efficiently prevent IFN production, and if this is partly responsible for increased pathogenicity in the current outbreak, remains elusive. Collectively, future identification of the molecules and signaling pathways targeted by SARS-CoV-2 will provide essential information on key antiviral modalities, which may also be those defective in patients with genetic predisposition to severe disseminated COVID-19.

## Adaptive Immune Responses to SARS-CoV-2

The cell-mediated immune response plays a critical role in antiviral immunity, and developing early and robust CD8^+^ and in particular, CD4^+^ T cell responses correlates well with positive outcomes during SARS-CoV infection (108, 109). Clinical investigations of severe COVID-19 patients consistently report neutrophilia and lymphopenia, with significantly depressed CD4^+^ T cell counts and decreased IFN-γ expression, as well as reduced numbers of regulatory and memory T cells (37, 110, 111). In addition, increased levels of plasma pro-inflammatory cytokines such as Interleukin (IL)-1β, IL-6, IL-8, and tumor necrosis factor-α (TNF-a) are observed in severe COVID-19 patients, indicative of a cytokine storm and subsequent ARDS development (32, 73). In support of these data, a study of 522 hospitalized COVID-19 patients reported a negative correlation between T cell numbers and serum IL-6, IL-10 and TNF-α levels (112). Furthermore, COVID-19 patients displayed increased expression of the T cell exhaustion markers, programmed cell death protein-1 (PD-1) and Tim-3, compared to healthy controls, suggesting that T cell survival and activation may play a role in protecting the host from severe SARS-CoV-2 development (112, 113). Studies investigating T cell phenotypes in asymptomatic or convalescent COVID-19 patients have been more limited. However, a recent study (114) identified circulating SARS-CoV-2-specific CD8^+^ and CD4^+^ T cells in ~70% and 100% of recovered COVID-19 patients, respectively. Importantly, this study reported the presence of SARS-CoV-2-reactive CD4^+^ T cells in healthy donors recruited in 2015–2018 (114). These data suggest that there is a cross-reactive coronavirus T cell memory response, but whether such pre-existing immunity influences clinical outcomes remains to be determined.

Cytotoxic T cells are important for clearing respiratory viruses and providing long-term protection; however, the magnitude of this response must be well-controlled to prevent pathological consequences (74, 115). Histological examination of a patient who died from severe SARS-CoV-2 infection identified that whilst the overall peripheral CD4^+^ and CD8^+^ T cell counts were significantly reduced, alveolar CD8^+^ T cells harbored high concentrations of cytotoxic granules, which may have contributed to severe tissue injury (116). Moreover, lung biopsy revealed desquamation of pneumocytes, pulmonary oedema, and hyaline membrane formation, indicative of early-stage ARDS development (116). On the other hand, a robust CD8^+^ T cell response is known to be important in SARS-CoV infection, and mild cases of COVID-19 have increased clonal expansion of CD8^+^ T cells, compared to severe cases (117). The pathological features of COVID-19 resemble those of SARS and MERS, both of which are thought to be largely caused by immune dysregulation rather than direct pathology induced by high viral load (111, 118), with a similar picture emerging for SARS-CoV-2.

A considerable number of studies have demonstrated that infection with SARS-CoV-2 initiates an antibody response. On average, seroconversion is observed between 10 and 14 days post infection, a timeline similar to that observed for the previous SARS-CoV epidemic (58, 119–123). Serological investigations of 262 hospitalized COVID-19 patients across the Chongqing region, China, reported simultaneous or sequential seroconversion (IgM and IgG) against recombinant antigens containing the nucleoprotein and a peptide from the spike protein of SARS-CoV-2 (124). Interestingly, IgG titers were higher in patients with severe COVID-19, compared to the non-severe group (124). A number of studies have also reported earlier or increased antibody titers in critical vs. non-critical COVID-19 patients, possibly reflecting more severe and invasive infection and thus making it difficult to determine the specific nature of the association (124, 125) whereas others report no correlation between disease severity and serum antibody levels (126). Such discrepancies between findings may be explained not only by the relatively small number of individuals investigated per study, but also due to the fact that different assays are used in the different studies, likely causing significant sensitivity and specificity challenges (123). Importantly, however, SARS-CoV-2 isolated from critically ill patients can be neutralized by sera from several patients, a finding that gives weight to the rapid development of therapeutic neutralizing antibodies (NAbs) as a potential means to control the current pandemic (58).

The possibility of re-infection with SARS-CoV-2 is a key area of investigation. Individuals who recovered from SARS-CoV or MERS-CoV infection displayed high tires of antibodies which waned after 2–3 years (127, 128). However, a small study of only three SARS-recovered individuals identified SARS-specific memory T cells 11 years after infection (129). Finally, the efficacy and longevity of immunity to SARS-CoV-2 is highly dependent upon the rate by which the virus alters its composition, i.e., the mutation rate. So far, studies suggest a relatively low virus mutation rate with mutations randomly spread throughout the viral genome, suggesting absence of positive selection toward increased virulence (60). Moreover, previous studies suggest that a virus crossing the species barrier is more likely to lose virulence over time than the opposite (130).

## Human Inborn Errors of Immunity Predisposing to Coronavirus Infection

Several predisposing factors are known to increase the risk of progression to severe COVID-19 (37). However, there is currently no explanation as to why younger individuals with no co-morbidities in rare cases have developed life-threatening COVID-19. Human inborn errors of immunity can alter the course of various viral infections (131), but in the case of disease progression in SARS-CoV-2 infection, little is known regarding the influence of the host genetic makeup. However, any genetic variant that results in a dysregulated or exaggerated immune response may contribute to lung immunopathology leading to life-threatening clinical manifestations.

It has been proposed that deficient type I IFN production may undermine the innate immune response during early SARS-CoV infection, resulting in a more severe disease course (80, 132, 133). Population studies have identified single nucleotide polymorphisms (SNPs) in the IFN-inducible genes *OAS1* and *MX1*, associated with susceptibility to SARS-CoV infection and disease progression (134, 135). Moreover, exogenous administration of type I IFN has been shown to inhibit SARS-CoV replication both *in vivo* (96, 99) and *in vitro* (95, 97, 100). Together, these data highlight the importance of an intact type I IFN pathway in the innate response to SARS-CoV, and it can be reasoned that any genetic mutations that impair this response may somehow predispose to COVID-19 disease progression. Consequently, it is worth considering that clinical outcome may be influenced by mutations of the PRRs and immune signaling pathways involved in recognition of SARS-CoV-2, including RIG-I, MDA5, and TLR3 and downstream IRF3 and IRF7, as well as molecules involved in effector function of type I and III IFN, such as IFNAR1/2 or the janus kinase (JAK) - signal transducer and activator of transcription (STAT) signaling pathways.

Emerging evidence suggests that activation of the complement system contributes to the pathogenesis of SARS-CoV-2 lung pathology (136, 137). Inhibition of the terminal complement pathway by targeting complement protein 5 (C5) has been proposed as an effective therapeutic intervention in CoV-mediated disease (138). Mannose-binding lectin (MBL) is a pattern recognition molecule, which binds to specific carbohydrate structures on the microbial surface, thereby activating complement terminal pathways (139). MBL serum immunodeficiency has been suggested to play a role, although controversial, in increased susceptibility to a wide range of viral and bacterial infections, mainly in children (140–142). A single study has identified that individuals carrying a low MBL-producing haplotype YB have an increased risk of acquiring SARS-CoV (143). Collectively, the precise role of the complement system as either having a detrimental or protective role in COVID-19 pathogenesis needs further study.

Within adaptive immunity, the impact of PIDs associated with antibody deficiencies and combined immunodeficiencies on SARS-CoV-2 outcomes are not fully clarified. However, some important information may be retrieved from a description of severe lung pathology and COVID-19 disease course in patients with common variable immunodeficiency (CVID) compared to patients with pure agammaglobulinemia (144). Although the results were based on few patients, it was noted that patients with agammaglobulinemia experienced relatively mild disease. However, patients with similarly low immunoglobulin levels but immune dysregulation as part of CVID experienced more severe disease, suggesting a role of immune dysregulation and abnormal B immune cell phenotype and potentially excessive IL-6 production in COVID-19 pathogenesis, although T cell deficiency may also be involved (144). Collectively, results of studies into COVID-19 disease presentation and progression in patients with known PIDs are eagerly awaited. Such data may facilitate understanding of what constitutes an inadequate immune response toward SARS-CoV-2 and from this knowledge determine the molecular and cellular correlates of protective immune responses and immunity in COVID-19.

As to the role of T cells in protection from severe COVID-19, it would be expected that T cell lymphopenia from any source might represent an increased risk of severe viral infection. However, not much evidence suggests that patients with primary T cell defects nor HIV infection are at a significantly elevated risk. When large cohort studies of SARS-infected and MERS-CoV-infected patients from previous epidemics were reviewed, HIV infection was not identified as an independent risk factor for infection by these coronaviruses (145, 146). However, evidence for the role of T cell defects on prevalence and severity of COVID-19 is scarce, and it is therefore too early to draw any conclusions (147).

Based on insight into immunopathogenesis and pathology during COVID-19, potential susceptibility genes may be involved in mechanisms of immune dysregulation, auto-inflammation or autoimmunity, thus involving the gain-of-function or loss of inhibition of various genes and pathways in cytokine and TLR signaling cascades, especially those affecting IL-1 and IL-6 synthesis and production (48–50). Likewise, genetic variants in endothelial cell biology and regulation may be anticipated to aggravate coagulopathy and thrombotic events. Specific evidence on such associations remains to be reported but should be part of searches in large unbiased whole exome sequencing approaches, such as the worldwide consortium (148) (covidhge.com).

Genetic variants in the major histocompatibility complex antigen loci (HLA) are well-known to influence host susceptibility to infectious disease (149). So far, only a limited number of studies have looked for an association between HLA haplotypes and genetic susceptibility or resistance to SARS coronavirus infection. However, an impact of HLA haplotypes may identify some of the unexplained differences in disease severity and mortality observed across different countries and different population ethnicities (150–154). Allele typing of 37 probable SARS patients in Taiwan identified an association between HLA-B^*^4601 and SARS-CoV infection (155); however, this was not confirmed in a study of 90 serologically confirmed SARS-CoV patients in Hong Kong (156, 157). The latter study instead identified an association between the HLA-B^*^0703 and HLA-DRB1^*^0301 genotypes and the development of SARS (157). In terms of the current pandemic, we might predict that genetic variations in the HLA molecules resulting in decreased binding specificities for SARS-CoV-2 peptides may confer a more severe COVID-19 disease course (158). Zhao et al. (159) investigated the relationship between ABO blood group and COVID-19 susceptibility in 2,173 Chinese patients. Interestingly, being of blood group A represented a higher risk for developing COVID-19 disease, whilst having blood group O was associated with a lower infection risk. A similar risk pattern for ABO blood groups has also been reported for the 2003 SARS-CoV outbreak (160); however, the exact mechanism of how blood group antigens might affect susceptibility remains to be clarified.

Genetic studies have identified polymorphisms in the *IL28B* gene which have been linked to hepatitis C virus (HCV) clearance (161). The product of this gene is type III IFN (IFN-λ). Given that genetic variants in IFN-λ appear to play an important role in the natural course of disease and severity during infection with HCV, it will be interesting to investigate if polymorphisms in the *IL28B* gene could also predispose to severe SARS-CoV-2 infection. Perhaps the most compelling reason to believe that genetic differences may account for the vast diversity in COVID-19 symptoms is a recent preprint study by British researchers who, using a mobile symptom tracker, recorded data from over 2.7 million app users. Analysis of same sex adult twins (*n* = 2,000^+^) showed that genetic factors accounted for 50% of the difference in COVID-19 symptoms (162). Although the results from the latter studies will need to be verified, these data highlight how genetic differences may explain the great variability in SARS-CoV-2 infection outcome.

As we start to gain further insight into the pathogenesis of severe COVID-19 and appreciate the major role played by inflammation and immunopathology, this raises the question as to whether there might be an increased risk of developing severe disease in certain individuals, and whether genes predisposing to those conditions should be investigated in a genetic approach. Such genes include *NLRP1, NLRP3, CASP1, MEFV*, and many others, which all encode proteins involved in inflammasome activation (163). Focus should also be on known genetic causes of hemophagocytic lymphohistiocytosis (HLH), including defects in the genes *PRF1, UNC13D, STX11, STXBP2, LYST, RAB27A* (164) either in the well-recognized homozygous forms, or possibly in heterozygous forms that may still predispose to HLH in the presence of a trigger, such as SARS-CoV-2 (165). However, the precise pathogenesis of macrophage activation syndrome and Kawasaki-like disease/PIMS in COVID-19 is not fully understood and may involve autoinflammation, autoimmunity, and/or immunopathology induced directly by the virus or as a secondary result of crossreactivity.

At the other end of the disease spectrum, there is the possibility that some individuals may avoid infection due to protective tissue types or gene mutations. The most prominent example to mention is the potential protective role of ACE2 defects, rendering cells restistant to SARS-CoV-2 infection, in analogy to protection from HIV infection by Delta32 *CCR5* homozygosity (166). Given the important role of host ACE2 in SARS-CoV-2 infection, we might speculate that *ACE2* genetic polymorphisms may be present in certain individuals, which could be exploited by SARS-CoV-2 and lead to severe clinical disease. Equally, *ACE2* polymorphisms may even offer some level of resistance. Indeed, one study has identified multiple rare genetic variants in *ACE2*, which are predicted to modify virus-host interaction and alter susceptibility to SARS-CoV-2 (167). Another study has also reported rare, genetic variants in *TMPRSS2* as possible disease modulators of SARS-CoV-2 infection in Italy (168). Although these findings necessitate functional validation, it gives weight to the hypothesis that rare, genetic mutations may explain COVID-19 disease severity.

## Therapeutic Developments and Trials

No therapeutics have yet been approved to treat diseases caused by SARS-CoV-2 or other coronaviruses (169, 170). By mid March 2020, WHO launched the global “Solidarity” clinical trial for COVID-19 treatments to assess the effectiveness of four potential therapeutic options, namely remdesivir, lopinavir/ritonavir with and without IFN-β, and chloroquine or hydroxychloroquine (171). Remdesivir, already early suggested to be the most promising compound (172, 173), is a pro-drug of an adenosine nucleotide analog, which upon incorporation in the growing RNA chain potently inhibits RNA-dependent RNA polymerases inducing synthesis arrest (172, 174). Remdesivir has broad antiviral activity against several RNA viruses *in vitro*, including SARS-CoV-2 (174–177), and *in vivo* against SARS-CoV and MERS-CoV (176, 178, 179). Whereas no significant improvement was observed in an initial trial of 237 patients receiving remdesivir (180), preliminary results from the larger Adaptive Covid-19 Treatment Trial (ACTT) showed that remdesivir significantly shortened the time to recovery in patients with lower airway involvement, and interestingly, another study found no difference between treatment with remdesivir for 5 days compared to 10 days (181, 182). However, importantly, remdesivir did not show major efficacy in patients with severe COVID-19 in the ICU (183), possibly because host responses may be major determinants at that stage, rather than viral replication. Promising results in controlling SARS-CoV-2 *in vitro* have also been demonstrated for the protease inhibitor lopinavir/ritonavir, licensed for HIV treatment (184, 185), as well as for chloroquine and hydroxychloroquine presently used in the treatment of malaria and autoimmune diseases (175, 186, 187). However, a randomized trial including 199 COVID-19 patients showed no immediate *in vivo* benefits of lopinavir/ritonavir (188). Similarly, clinical trials on chloroquine and hydroxychloroquine have identified methodological limitations and conflicting results, even suggesting a negative effect of hydroxychloroquine (189–195). In May 2020, a multinational analysis reported the use of hydroxychloroquine or chloroquine, with or without a macrolide, for treatment of COVID-19 comprising data from 671 hospitals in six continents (196). This study failed to show any beneficial effect of the two drugs; moreover, concerns were raised about whether these drugs caused higher in-hospital mortality (197). Notably, the study was retracted shortly after publication, whereas the hydroxychloroquine studies currently continue after evaluation of the safety data by the WHO (198).

Agents which target the host are also potentially valuable treatment options. Interestingly, the agent camostat mesylate has been reported to block the cellular serine protease TMPRSS2, thereby inhibiting SARS-CoV-2 entry into host cells (67), and increases survival rates ~60% in an *in vivo* SARS-CoV model (199). Camostat mesylate is widely used in Japan for chronic pancreatitis and postoperative reflux esophagitis (200, 201), and currently, a clinical trial in Denmark investigating the impact of this compound on COVID-19 is in its recruitment stage (202).

The effectiveness of agents blocking either IL-1 or IL-6, i.e., the receptor-targeted monoclonal antibodies anakinra and tocilizumab, respectively, in the treatment of patients with severe COVID-19 in the ICU have received considerable interest due to the well-established role of these cytokines in immunopathology and association with poor clinical outcome (32, 63). Promising results have been reported for individuals or small groups of COVID-19 patients treated with tocilizumab (203, 204) and anakinra (205, 206), the latter potentially also beneficial in treating COVID-19 related HLH (207). The results of ongoing randomized, controlled clinical trials (50, 208, 209) will ultimately determine whether such strategies to dampen immune responses will prove beneficial and safe. So far, no significantly increased tendency to severe COVID-19 was reported in patients receiving these medications (DMARDs) for the treatment of rheumatologic conditions (210). On a more hypothetical basis, the complement system has been suggested as a target for treatment (211). Finally, numerous ongoing studies are investigating additional novel therapeutic strategies (212) and candidate vaccines (213) against SARS-CoV-2. Despite the rapidly evolving number of publications on potential COVID-19 therapeutics, thus far only a very limited number of well-designed clinical trials are available. However, to evaluate the effectiveness and potential adverse effects of future treatments, the results of thorough large-scale, randomized, controlled trials are crucial. Notably, a number of fundamental immunological and virological questions need to be addressed before a vaccine candidate is available. These include aspects related to the mutation rate of the virus, the strength of immunity induced by SARS-CoV-2 and the relative contributions from humoral or cellular immunity, and not least the duration of a protective immune response.

## Concluding Remarks

The current global COVID-19 pandemic is a major challenge for all involved in studies of the role of human genetics in the face of a novel infectious disease. Sequencing children and young or middle-aged individuals with severe COVID-19, who are otherwise healthy, will identify rare, deleterious mutations that lead to SARS-CoV-2 infection and severe clinical outcome. This will offer unique insights on the disease pathogenesis *in natura*, which will undoubtedly offer new therapeutic potentials (148). Coronaviruses appear at regular intervals, and understanding the molecular mechanisms of COVID-19 development is imperative not only to ending the current pandemic, but also for controlling future potential outbreaks.

## Author Contributions

TM conceived the idea, all authors contributed to the first version of the manuscript. MC-T and TM revised and finalized the manuscript and all co-authors read, corrected, and approved the final version of the manuscript. TM prepared Figure 1, whereas MC-T prepared Figure 2 with input from co-authors.

## Conflict of Interest

The authors declare that the research was conducted in the absence of any commercial or financial relationships that could be construed as a potential conflict of interest.
